# Surgical Approaches for Primary Gastric GIST After the Introduction of Robotic Surgery: A 10-Year Single-Center Experience

**DOI:** 10.3390/cancers18162562

**Published:** 2026-08-10

**Authors:** Julia Michel, Jens Hoeppner, Michael Leitz, Fabian Nimczewski, Zsolt Madarasz

**Affiliations:** Department of Surgery, Medical School and University Medical Center OWL, Bielefeld University, Campus Hospital Lippe, 32756 Detmold, Germany; jens.hoeppner@uni-bielefeld.de (J.H.); michael.leitz@klinikum-lippe.de (M.L.); fabian.nimczewski@klinikum-lippe.de (F.N.); zsolt.madarasz@klinikum-lippe.de (Z.M.)

**Keywords:** gastrointestinal stromal tumor, gastric GIST, robotic surgery, laparoscopic surgery, minimally invasive surgery, surgical oncology

## Abstract

Background/Objectives: Robotic surgery offers an additional minimally invasive option for gastric gastrointestinal stromal tumor (GIST) resection, particularly in anatomically demanding locations. Evidence regarding its integration into routine surgical practice remains limited. This study evaluated the evolution of surgical approaches for primary localized gastric GIST following the introduction of an institutional robotic program. Methods: We retrospectively analyzed 44 consecutive patients who underwent curative-intent resection for primary localized gastric GIST at a tertiary referral center between July 2015 and June 2025. Surgical management and perioperative outcomes were described for an early era (2015–2021; *n* = 29) and a later era after the introduction of robotic surgery (2022–2025; *n* = 15). Results: Overall, 22 procedures were completed laparoscopically, six robotically, and 13 through a primary open approach; three laparoscopic procedures were converted to open surgery. In the later era, robotic surgery accounted for 40.0% of resections, and no conversions occurred. The proportion of completed minimally invasive resections was 62.1% in the early era and 66.7% in the later era. R0 resection was achieved in all patients, and no tumor rupture was documented. Median operative time was 73.5 min, median postoperative length of stay was 7.5 days, and major morbidity occurred in six patients (13.6%). One patient (2.3%) died from sepsis within 30 days after reoperation for a postoperative gastric suture-line leak. Conclusions: Following the introduction of robotic surgery, management of primary gastric GIST evolved toward a more diversified and individualized surgical strategy. Robotic surgery was incorporated as an additional minimally invasive option, while the overall proportion of completed minimally invasive resections remained stable. These findings represent an early institutional experience and do not establish equivalence or comparative superiority among surgical approaches.

## Simple Summary

Gastric gastrointestinal stromal tumors are rare tumors for which surgery is the main treatment when the disease is localized. Laparoscopic and open procedures are established, while the role of robotic surgery in routine clinical practice remains less clear. We analyzed 44 consecutive patients treated with curative intent over 10 years at a tertiary referral center and examined how surgical strategies changed after the introduction of robotic surgery. Six patients underwent robotic resection, accounting for 40% of operations in the later study period. The overall proportion of completed minimally invasive procedures remained stable, complete tumor removal was achieved in all patients, and no tumor rupture was recorded. These findings suggest that robotic surgery became an additional minimally invasive option for selected gastric gastrointestinal stromal tumors and diversified the distribution of minimally invasive approaches without increasing the overall minimally invasive surgery rate. Larger multicenter studies are needed to define its comparative role.

## 1. Introduction

Gastrointestinal stromal tumors (GIST) are the most common mesenchymal neoplasms of the gastrointestinal tract, with approximately 60% arising in the stomach [1]. Their biological behavior ranges from indolent lesions with very low malignant potential to highly aggressive tumors with metastatic capability [1,2,3]. Surgical resection remains the standard treatment for localized gastric GIST, aiming to achieve complete (R0) resection while avoiding tumor rupture, which is a major adverse prognostic factor associated with peritoneal dissemination and recurrence [4,5,6]. Unlike gastric adenocarcinoma, routine lymphadenectomy is generally unnecessary because lymphatic spread is rare. Risk stratification and perioperative management are guided by tumor size, anatomical location, mitotic activity, molecular characteristics, and the selective use of perioperative imatinib according to current international guidelines [1,4,5]. Several validated risk classifications, contour maps, and prognostic models have identified tumor size, mitotic activity, anatomical site, and tumor rupture as major determinants of recurrence after complete resection [7,8,9]. Randomized trials established the benefit of adjuvant imatinib after complete resection and demonstrated improved long-term outcomes with three years rather than one year of treatment in high-risk disease [10,11,12]. Neoadjuvant imatinib may additionally facilitate complete and organ-preserving resection in selected patients with large or anatomically challenging gastric GIST [13].

Over the past two decades, minimally invasive surgery has become an established treatment option for appropriately selected gastric GIST. Laparoscopic resection has demonstrated favorable perioperative outcomes while maintaining oncologic safety when fundamental surgical principles are respected [5,14,15]. These findings are supported by systematic reviews, prospective and propensity-matched cohorts, and large single-center series, including selected patients with tumors larger than 5 cm [16,17,18,19,20]. However, minimally invasive surgery remains technically demanding for tumors located close to the gastroesophageal junction or pylorus, on the posterior gastric wall, or in cases requiring complex intracorporeal reconstruction or precise organ-preserving resection. In these situations, robotic surgery offers several technical advantages, including enhanced dexterity, three-dimensional visualization, improved ergonomics, and advanced intracorporeal suturing capabilities, which may facilitate minimally invasive treatment in anatomically challenging cases [21,22,23].

Although robotic surgery has become increasingly integrated into upper gastrointestinal surgery, evidence specifically addressing robotic resection of gastric GIST remains limited. Most available studies consist of small institutional series, multicenter retrospective cohorts, database analyses, or meta-analyses based on non-randomized data and primarily compare short-term perioperative outcomes between surgical approaches [21,22,23,24,25,26,27,28,29,30]. Much less is known about how robotic surgery is implemented within routine clinical practice, how it changes institutional treatment strategies over time, and how it complements rather than replaces established laparoscopic and open techniques in the management of this relatively uncommon disease.

The introduction of a robotic surgical program represents more than the adoption of a new operative platform; it may fundamentally alter institutional decision-making regarding patient selection and the choice of surgical approach. For rare indications such as gastric GIST, describing this process may provide valuable real-world information for centers implementing robotic surgery and help place early institutional experience into the context of current surgical practice.

Therefore, the aim of the present study was to evaluate the evolution of surgical management for primary localized gastric GIST over a 10-year period at a tertiary referral center, with particular emphasis on the implementation of robotic surgery and its integration into an individualized surgical strategy. Rather than comparing surgical techniques, this study describes how robotic surgery became incorporated into routine clinical practice while evaluating whether the evolution of surgical approaches remained consistent with established oncological principles, including complete resection and avoidance of tumor rupture.

## 2. Materials and Methods

### 2.1. Study Design and Patient Selection

This retrospective single-center cohort study is based on an institutional GIST database and included patients treated between July 2015 and June 2025. Patients with non-gastric, multifocal, extra-gastrointestinal, or unknown primary GIST were excluded from the main gastric GIST cohort. Robot-assisted procedures involving non-gastric GIST were considered only in the exploratory contextual analysis of institutional robotic activity.

Patients in whom gastric GIST was identified histologically or resected during major surgery performed primarily for another disease were not considered GIST-directed surgical cases. These patients were excluded from the main cohort and perioperative outcome analysis because operative time, postoperative morbidity, and length of hospital stay could not be attributed specifically to the GIST procedure. Robot-assisted procedures from this group remained eligible for the exploratory contextual analysis. Minor or clearly separable concomitant procedures performed during GIST-directed surgery were retained in the cohort and documented separately. This applied to one patient who underwent synchronous cholecystectomy.

The main analysis was restricted to patients with primary localized gastric GIST who underwent curative-intent resection. Patients with metastatic disease at the time of surgery or those undergoing palliative resection were excluded from the main cohort. The detailed cohort selection process is presented in Table S1.

The study period was divided into an early era from July 2015 to December 2021 and a later era from January 2022 to June 2025, during which robotic gastric GIST surgery was introduced at our institution. The choice of surgical approach was based on tumor size, anatomical location, growth pattern, anticipated technical complexity, surgeon judgment, and availability of the robotic platform. Robotic surgery was considered when the platform was available and a minimally invasive robotic approach appeared anatomically and technically feasible. For the six robotic gastric GIST resections, tumor location, pathological tumor size, growth pattern, type of gastric resection, and the anatomical or technical context of operative planning were additionally extracted from preoperative imaging, endoscopic or endosonographic findings, multidisciplinary documentation, operative reports, and pathology reports.

### 2.2. Data Collection and Definitions

Clinical, operative, pathological, and postoperative data were extracted retrospectively from electronic medical records, operative reports, pathology reports, and institutional documentation systems. Variables included age, sex, body mass index (BMI), American Society of Anesthesiologists (ASA) classification, Eastern Cooperative Oncology Group (ECOG) performance status, tumor size, gastric tumor location, mitotic activity, mutation status, preoperative biopsy, incidental detection, neoadjuvant and adjuvant systemic therapy, surgical approach, type of resection, resection status, tumor rupture, operative time, length of hospital stay, postoperative complications, reinterventions, reoperations, and 30-day mortality. Operative time was defined as the time from skin incision to skin closure. Incidental detection was defined as identification of a gastric GIST during diagnostic evaluation or treatment for an unrelated condition. Patients were eligible for the main cohort only if they subsequently underwent GIST-directed curative-intent resection and met the remaining inclusion criteria. Postoperative complications occurring within 30 days after surgery were classified according to the Clavien–Dindo classification [31]. Major complications were defined as Clavien–Dindo grade III or higher.

### 2.3. Study Outcomes

The primary outcome was the distribution of the final surgical approach in the early and later eras. Secondary outcomes included conversion to open surgery, R0 resection, tumor rupture, operative time, length of hospital stay, postoperative morbidity, major morbidity, 30-day reoperation, and 30-day mortality.

As an exploratory contextual analysis, all robot-assisted procedures involving GIST after introduction of the institutional robotic program were identified from the institutional database and summarized by year, primary site, and clinical context. Synchronous procedures performed during major surgery for another disease and procedures involving non-gastric GIST were not included in the perioperative outcome analysis of the main cohort.

The final surgical approach was categorized as completed laparoscopic, completed robotic, primary open, or conversion to open surgery. Conversion was defined as any procedure initially started laparoscopically that required open completion. Reasons for conversion were extracted from the operative reports and categorized as anatomical location, tumor morphology, technical limitation, bleeding, or other causes. Gastric tumor location was categorized as proximal stomach (cardia or fundus), gastric body, distal stomach (antrum or pylorus), or multiple, overlapping, or unspecified location. The type of gastric resection was categorized as local excision or wedge resection, segmental or partial gastric resection, distal or subtotal gastrectomy, or total, proximal, or esophagogastric resection. R0 resection was defined as microscopically margin-negative resection.

Tumor rupture was defined as intraoperative or spontaneous disruption of the tumor capsule with potential tumor cell dissemination. Mitotic activity was extracted from the original pathology reports. Earlier reports expressed mitotic activity per 50 high-power fields (HPF), whereas more recent reports used a standardized area of 5 mm^2^, reflecting a change in institutional pathology reporting practice. Values were retained in the unit reported in the original pathology report and categorized as ≤5 versus >5 mitoses without mathematical conversion. Because the microscope field area represented by 50 HPF in the historical reports was not available retrospectively, equivalence between 50 HPF and 5 mm^2^ could not be verified.

Armed Forces Institute of Pathology (AFIP) risk categories were assigned according to the criteria proposed by Miettinen and Lasota, incorporating tumor site, tumor size, and the mitotic threshold reported in the corresponding pathology report [1]. For presentation, the categories “none” and “very low” were combined because of the limited cohort size. AFIP risk classification was considered not assessable in patients who had received neoadjuvant imatinib, as post-treatment tumor size and mitotic activity may not reliably reflect untreated biological risk. Because the microscope field area represented by 50 HPF in the historical reports could not be reconstructed, some risk-category misclassification cannot be excluded.

Mutation status was recorded when available from the original molecular pathology reports. Because molecular testing was not performed systematically throughout the entire study period, the availability of molecular testing was reported descriptively, and mutation subtype distributions were not compared between the early and later eras.

### 2.4. Statistical Analysis

Continuous variables are presented as median and interquartile range (IQR), while categorical variables are presented as absolute numbers and percentages. Owing to the limited cohort size and the exploratory, descriptive purpose of the study, no formal hypothesis testing was performed. Differences between the early and later eras were interpreted descriptively. No multivariable regression analysis was conducted. Data management and descriptive statistical analyses were performed using Microsoft Excel for Microsoft 365 (Microsoft Corporation, Redmond, WA, USA).

### 2.5. Ethics Approval 

The study was conducted in accordance with the Declaration of Helsinki and was approved by the Ethics Committee of the Medical Association of Westphalia-Lippe and the University of Münster (reference no. 2026-074-f-S; decision dated 20 March 2026). The committee confirmed that the project constituted a retrospective analysis of existing clinical treatment data addressing a predefined research question. The requirement for individual informed consent was waived.

### 2.6. Use of Generative Artificial Intelligence

During the preparation of this manuscript, the authors used ChatGPT (OpenAI, https://chatgpt.com/, accessed on 6 August 2026) to support English-language editing and manuscript restructuring. The tool was not used for data collection, statistical analysis, or interpretation of the results. All output was critically reviewed and edited by the authors, who take full responsibility for the content of the publication.

## 3. Results

### 3.1. Patient Cohort

**Figure 1:**
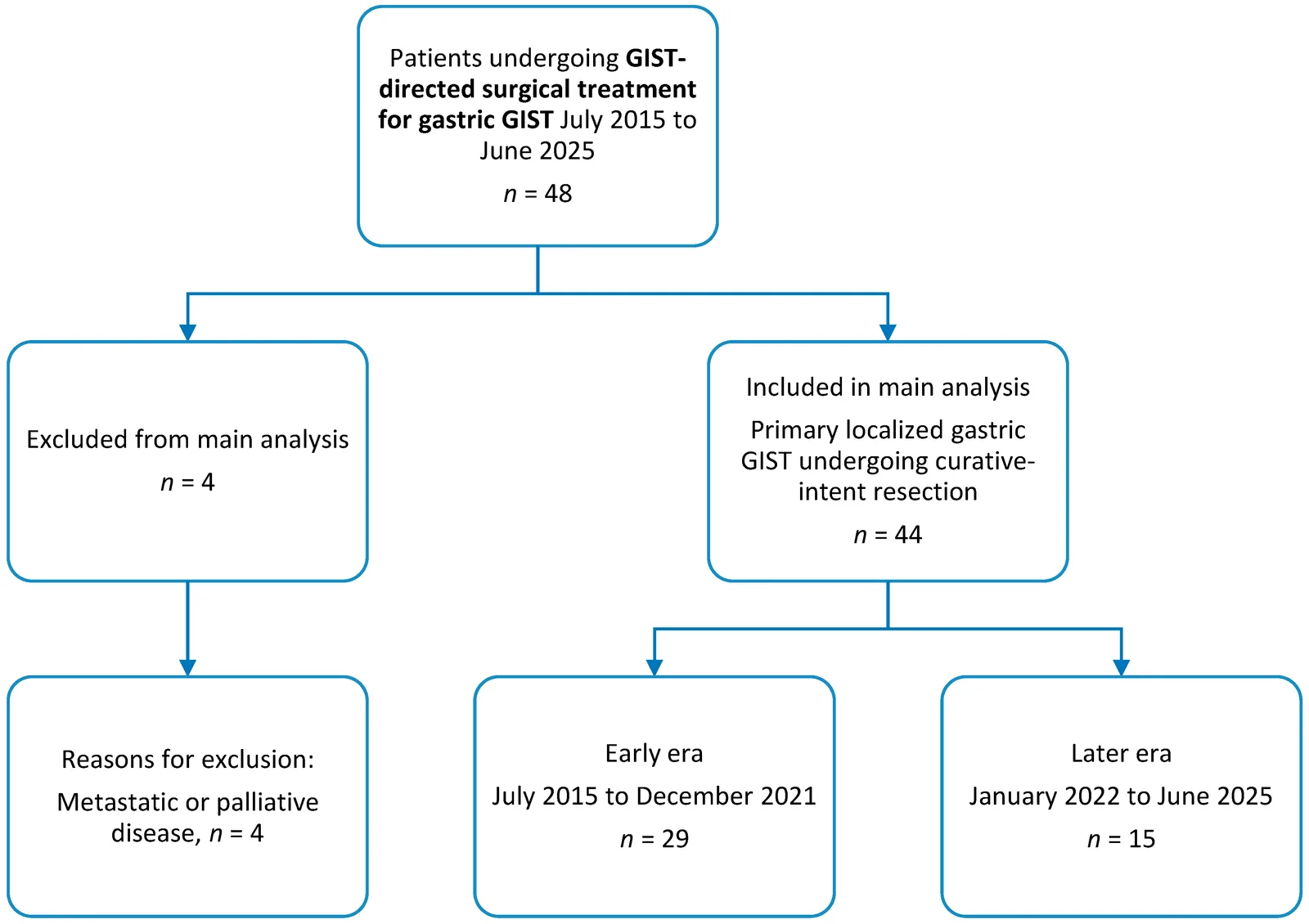
Study flowchart. Of 48 patients undergoing GIST-directed surgical treatment for gastric GIST between July 2015 and June 2025, four patients with metastatic or palliative disease were excluded, leaving 44 patients with primary localized gastric GIST undergoing curative-intent resection for the main analysis.

A total of 48 patients underwent GIST-directed surgical treatment for gastric GIST during the study period. Four patients with metastatic or palliative disease were excluded from the main analysis. The final main cohort therefore comprised 44 patients with primary localized gastric GIST undergoing curative-intent resection (Figure 1). The complete cohort selection process from the institutional GIST database is shown in Table S1.

The median age of the patients was 71 years (IQR 62.8–79.5), and 25 patients were female (56.8%). The median tumor size was 3.9 cm (IQR 2.5–5.1). Tumors were most frequently located in the gastric body (22 patients, 50.0%), followed by the distal stomach (12 patients, 27.3%) and the proximal stomach (8 patients, 18.2%). Two patients (4.5%) had multiple, overlapping, or insufficiently specified tumor locations. An ECOG performance status of 2 or higher was documented in 6 patients (13.6%). A mitotic count greater than 5 was documented in 7 patients (15.9%). According to the AFIP classification, 28 patients (63.6%) had no or very low risk, 6 patients (13.6%) had low risk, 4 patients (9.1%) had moderate risk, and 3 patients (6.8%) had high risk. AFIP risk classification was not assessable in 3 patients (6.8%) who had received neoadjuvant imatinib. Molecular testing results were available for 13 patients (29.5%). Because testing was not performed systematically throughout the study period, mutation subtypes were not compared between the two eras. Preoperative biopsy was performed in 15 patients (34.1%). Neoadjuvant imatinib was administered in 3 patients (6.8%), and adjuvant imatinib in 12 patients (27.3%). Patient, tumor, and treatment characteristics are summarized in Table 1.

### 3.2. Surgical Approach and Operative Characteristics

Completed laparoscopic resection was achieved in 22 patients (50.0%), robotic resection in 6 patients (13.6%), and primary open surgery in 13 patients (29.5%). Three laparoscopic procedures (6.8%) required conversion to open surgery. Conversions were prompted by unfavorable tumor location or morphology requiring an extended or technically more appropriate open gastric resection; none was caused by intraoperative bleeding or another surgical complication. Local excision or wedge resection was the most frequently performed procedure and was used in 32 patients (72.7%). Six patients (13.6%) underwent distal or subtotal gastrectomy, 3 patients (6.8%) underwent segmental or partial gastric resection, and 3 patients (6.8%) underwent total, proximal, or esophagogastric resection. Four patients (9.1%) underwent GIST-related multivisceral resection. One patient (2.3%) underwent synchronous cholecystectomy in addition to gastric GIST resection and was retained in the main cohort. The median operative time was 73.5 min (IQR 52.8–138.3). R0 resection was achieved in all patients, and no tumor rupture was documented. Surgical approach and operative characteristics are summarized in Table 2.

### 3.3. Perioperative Outcomes

The median length of hospital stay was 7.5 days (IQR 5–9). Postoperative complications occurred in 14 patients (31.8%), including 8 patients (18.2%) with Clavien–Dindo grade I–II complications and 6 patients (13.6%) with grade III or higher complications. One patient underwent reoperation for a postoperative gastric suture-line leak and subsequently died from sepsis within 30 days after surgery. Perioperative outcomes are summarized in Table 3, and patient-level details of postoperative complications are provided in Table S2.

### 3.4. Characteristics of the Robotic Gastric GIST Resections

The six robotic gastric GIST resections were heterogeneous with respect to tumor location, growth pattern, and the required extent of resection. Three tumors showed predominantly or presumed exophytic/extragastric growth, whereas three were predominantly intramural or endophytic. Resections ranged from local or wedge resection to segmental gastric resection and complex resection and reconstruction at the cardia. Case-level characteristics and the anatomical or technical context of operative planning are summarized in Table 4.

### 3.5. Distribution of Surgical Approaches

**Figure 2:**
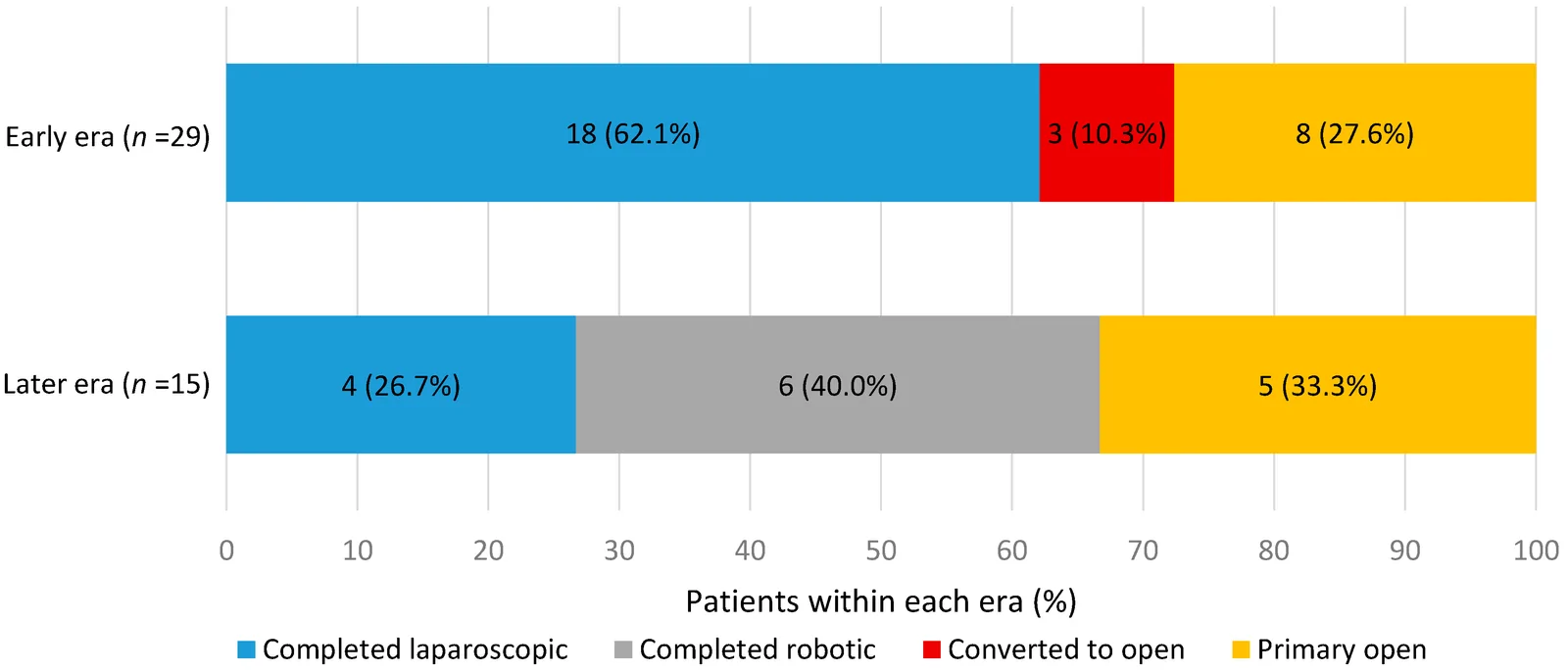
Final surgical approach in the early and later study eras. Bars show percentages within each era; labels indicate absolute numbers and percentages.

In the early era from 2015 to 2021, 29 patients underwent curative-intent resection for localized gastric GIST, compared with 15 patients in the later era from 2022 to 2025. Completed laparoscopic resections decreased from 18 of 29 patients (62.1%) in the early era to 4 of 15 patients (26.7%) in the later era, while robotic resections increased from 0 to 6 of 15 patients (40.0%). When completed laparoscopic and robotic procedures were considered together, the proportion of completed minimally invasive resections was 62.1% in the early era and 66.7% in the later era. Primary open surgery remained relevant in both periods, accounting for 8 of 29 procedures (27.6%) in the early era and 5 of 15 procedures (33.3%) in the later era. Conversion to open surgery occurred in 3 patients (10.3%) in the early era and in none of the patients in the later era (Table 2 and Figure 2).

Despite this change in surgical approach, R0 resection was achieved in all patients in both periods, and no tumor rupture was observed. Median length of hospital stay was 8 days in the early era and 6 days in the later era. Overall postoperative morbidity was 34.5% in the early era and 26.7% in the later era, while major morbidity was 13.8% and 13.3%, respectively (Table 3).

### 3.6. Institutional Robotic Activity Beyond the Main Cohort

**Figure 3:**
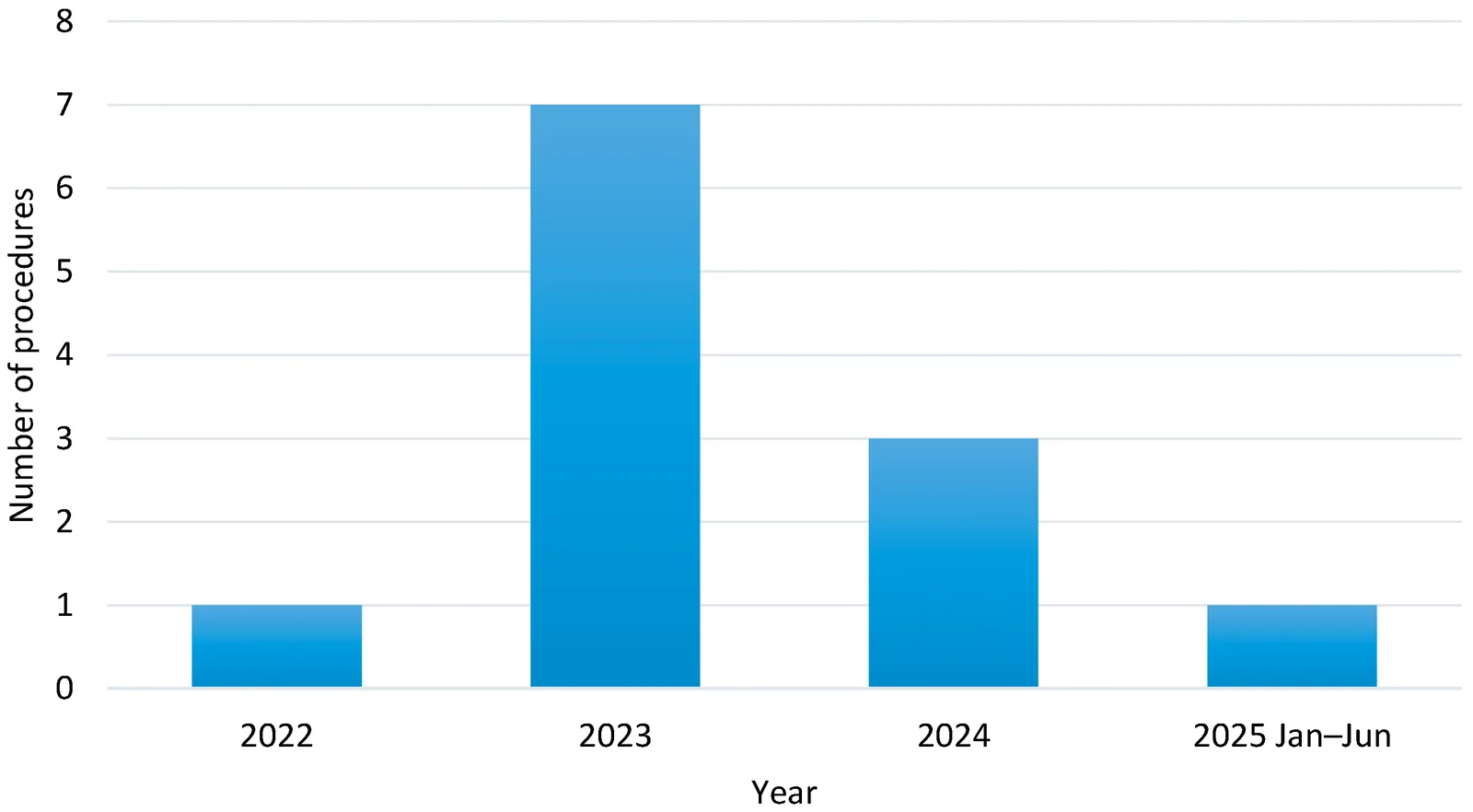
Annual robot-assisted procedures involving GIST after introduction of the institutional robotic program. Bars show the annual number of robot-assisted procedures involving GIST, and labels indicate absolute counts. Procedures included GIST-directed gastric resections, synchronous gastric GIST resections performed during major surgery for another disease, and non-gastric GIST procedures. The year 2025 includes procedures performed between January and June only.

After introduction of the robotic program in 2022, robot-assisted procedures involving GIST were performed in each subsequent study year. Overall, 12 robot-assisted procedures involved a GIST: 9 involved a gastric GIST and 3 involved a non-gastric GIST. Among the gastric cases, 6 were GIST-directed resections included in the main cohort and 3 were synchronous gastric GIST resections performed during major surgery for another disease. Annual totals were 1 procedure in 2022, 7 in 2023, 3 in 2024, and 1 between January and June 2025 (Table S3; Figure 3). Synchronous and non-gastric procedures were not included in the perioperative outcome analysis of the main cohort.

## 4. Discussion

The present study describes the early institutional implementation of robotic surgery for primary localized gastric GIST within a tertiary referral center over a 10-year period. Rather than comparing surgical approaches, the objective was to evaluate how the introduction of robotic technology influenced institutional decision-making and the overall surgical strategy for this uncommon disease. The principal finding was that robotic surgery became an additional component of minimally invasive gastric GIST management during the later study period, accounting for 40% of resections while the overall proportion of completed minimally invasive procedures remained stable. From an oncological perspective, this redistribution of approaches was not associated with compromised resection quality, as R0 resection was achieved in all patients and no tumor rupture was documented. At the same time, open surgery remained an indispensable option for anatomically complex tumors requiring more extensive gastric resection.

The introduction of robotic surgery should not be interpreted as an expansion of the overall minimally invasive surgery rate or as a replacement for conventional laparoscopy. During the later study period, laparoscopic procedures were complemented by robotic resections, whereas the combined proportion of completed laparoscopic and robotic operations remained largely unchanged. The principal observed change was therefore a diversification and redistribution of minimally invasive approaches according to tumor location, anticipated technical complexity, and reconstructive requirements. Such a strategy reflects contemporary surgical practice, in which multiple operative platforms coexist and are selected according to patient- and tumor-specific characteristics rather than institutional preference alone. Hybrid laparoscopic–endoscopic techniques have likewise been developed to minimize unnecessary gastric wall resection and preserve gastric function in selected submucosal tumors, illustrating the broader movement toward individualized organ-preserving surgery [32,33].

An additional oncological consideration is the interpretation of microscopic resection margins. Although R0 resection remains the intended surgical goal, the prognostic significance of an isolated R1 margin in GIST is less clear than that of macroscopic residual disease or tumor rupture. Available retrospective data suggest that the adverse outcomes observed after R1 resection may be driven partly by aggressive tumor biology and concomitant rupture, whereas R1 status without rupture has not consistently been associated with inferior recurrence-free or overall survival. Consequently, re-resection after an incidental R1 finding is not routinely indicated and should be considered selectively when it can be performed without substantial morbidity. In the present cohort, all resections were R0 and no tumor rupture occurred; therefore, our data do not permit an assessment of outcomes after R1 resection [34].

Our observations are consistent with the current literature suggesting that robotic surgery should be regarded as an additional minimally invasive option rather than a universal replacement for laparoscopic surgery. Published institutional series, multicenter cohorts, database analyses, and systematic reviews have reported broadly comparable short-term oncological and perioperative outcomes between robotic and laparoscopic approaches, while emphasizing the potential technical advantages of robotic systems in anatomically demanding gastric locations [21,22,23,24,25,26,27,28,29,30]. However, the descriptive similarity of selected outcomes across eras or surgical approaches in the present cohort should not be interpreted as evidence of equivalence. Treatment allocation was non-random and was likely influenced by tumor size, location, anatomical complexity, surgeon judgment, availability of the robotic platform, and institutional experience over time. Confounding by indication therefore precludes causal comparison of the operative approaches. Accordingly, our findings should be interpreted only as an account of how robotic surgery was selectively integrated into clinical decision-making while established oncological principles were maintained.

Given the rarity of gastric GIST and the small number of robot-assisted resections, the present series does not permit a formal assessment of an institutional or surgeon-specific learning curve. The robotic platform was used when it was available and when tumor location, growth pattern, and anticipated technical complexity suggested that a robotic minimally invasive approach was anatomically and technically feasible. Accordingly, the temporal distribution of robotic procedures should be interpreted as selective institutional adoption rather than as evidence of a continuous procedural learning curve. Increasing familiarity with the robotic platform may nevertheless have influenced operative planning and patient selection. Any potential effect of increasing familiarity with the robotic platform on perioperative outcomes cannot be distinguished from differences in case selection and case complexity; the limited number of cases and the absence of surgeon-level cumulative performance metrics further preclude quantification of such effects. Future prospective registries should capture case sequence, surgeon-level experience, procedural metrics, and the different clinical settings separately.

Despite its limitations, this study has several strengths. It represents a complete institutional experience covering the implementation phase of robotic surgery for a rare surgical indication over a 10-year period. All consecutive patients undergoing GIST-directed surgery for localized gastric GIST were included, providing a real-world overview of changes in surgical strategy within a tertiary referral center. Rather than focusing solely on robotic cases, the study reflects how robotic surgery was incorporated into routine clinical practice alongside established laparoscopic and open approaches.

This study has several limitations. First, its retrospective single-center design and limited sample size restrict the generalizability of the findings. In particular, only six robotic procedures were included in the main cohort, precluding reliable comparisons or claims of equivalence between robotic, laparoscopic, and open surgery. Because gastric GIST is a rare indication and only six robotic resections were included in the main cohort, a formal assessment of an institutional or surgeon-specific learning curve was not possible. Robotic procedures were performed selectively according to platform availability and anticipated anatomical and technical feasibility rather than according to a predefined stepwise implementation protocol. A separate case-specific rationale for selecting the robotic rather than laparoscopic approach was not documented prospectively; the anatomical and technical contexts presented for the robotic cases were reconstructed retrospectively from the available clinical documentation. In one patient, operative time included a synchronous cholecystectomy and could not be separated retrospectively from the duration of gastric GIST resection. Second, the choice of surgical approach was based on tumor size, anatomical location, technical complexity, surgeon judgment, and the availability of robotic surgery, resulting in an inherent risk of selection bias and confounding by indication. Third, clinical and pathological documentation evolved during the 10-year study period. In particular, mitotic activity was reported per 50 HPF in earlier pathology reports and per 5 mm^2^ in more recent reports. Although the corresponding AFIP risk tables commonly apply a threshold of five mitoses, the tissue area represented by 50 HPF depends on microscope optics and is not necessarily equivalent to 5 mm^2^. Because the historical microscope field areas could not be reconstructed, some risk-category misclassification cannot be excluded. Molecular testing was available only for a subset of patients and was not performed systematically throughout the study period. Therefore, mutation subtype distributions could not be reliably assessed or compared between the two eras. Fourth, patients with metastatic disease or undergoing palliative resection were excluded from the main curative-intent cohort; therefore, the R0 resection rate should be interpreted within this selected population. Finally, the present analysis focused on surgical and perioperative outcomes, and mature data on recurrence-free and overall survival were not available for a robust long-term oncological analysis.

## 5. Conclusions

Over the 10-year study period, surgical management of primary localized gastric GIST evolved toward an individualized strategy incorporating laparoscopic, robotic, and open approaches. Robotic surgery was incorporated as an additional minimally invasive option and diversified the distribution of minimally invasive approaches, but it did not increase the overall proportion of completed minimally invasive procedures or replace open surgery in complex cases. Within the curative-intent cohort, R0 resection was achieved in all patients, and no tumor rupture was documented. These findings describe selective institutional adoption and do not establish equivalence or superiority among surgical approaches. Larger multicenter studies are required to determine the comparative role of robotic surgery in gastric GIST.

## Figures and Tables

**Table 1:** Patient, tumor, and treatment characteristics of patients with primary localized gastric GIST.

Variable	Early Era 2015–2021 (*n* = 29)	Later Era 2022–2025 (*n* = 15)	Overall Cohort (*n* = 44)
**Patient characteristics**			
Age, years, median (IQR)	70 (63.0–81.0)	72 (62.5–77.0)	71 (62.8–79.5)
Female sex, *n* (%)	18 (62.1)	7 (46.7)	25 (56.8)
BMI, median (IQR)	26 (23–28)	27 (24.5–33.0)	26.5 (23–29)
ASA score, median (IQR)	3 (2–3)	2 (2–3)	2.5 (2–3)
ECOG ≥ 2, *n* (%)	5 (17.2)	1 (6.7)	6 (13.6)
**Tumor characteristics**			
Tumor size, cm, median (IQR)	3.7 (2.4–4.7)	4.4 (2.8–5.6)	3.9 (2.5–5.1)
Mitotic count > 5, *n* (%)	4 (13.8)	3 (20.0)	7 (15.9)
Gastric location, *n* (%)			
Proximal stomach (cardia/fundus)	6 (20.7)	2 (13.3)	8 (18.2)
Gastric body	15 (51.7)	7 (46.7)	22 (50.0)
Distal stomach (antrum/pylorus)	7 (24.1)	5 (33.3)	12 (27.3)
Multiple, overlapping, unspecified	1 (3.4)	1 (6.7)	2 (4.5)
AFIP risk category, *n* (%)			
None/very low	19 (65.5)	9 (60.0)	28 (63.6)
Low	4 (13.8)	2 (13.3)	6 (13.6)
Moderate	2 (6.9)	2 (13.3)	4 (9.1)
High	2 (6.9)	1 (6.7)	3 (6.8)
Not assessable after neoadjuvant therapy	2 (6.9)	1 (6.7)	3 (6.8)
**Diagnostic and treatment characteristics**			
Incidental detection, *n* (%)	2 (6.9)	7 (46.7)	9 (20.5)
Preoperative biopsy, *n* (%)	10 (34.5)	5 (33.3)	15 (34.1)
Molecular testing available, *n* (%)	10 (34.5)	3 (20.0)	13 (29.5)
Neoadjuvant imatinib, *n* (%)	2 (6.9)	1 (6.7)	3 (6.8)
Adjuvant imatinib, *n* (%)	7 (24.1)	5 (33.3)	12 (27.3)

Abbreviations: AFIP, Armed Forces Institute of Pathology; ASA, American Society of Anesthesiologists; BMI, body mass index; ECOG, Eastern Cooperative Oncology Group; GIST, gastrointestinal stromal tumor; HPF, high-power field; IQR, interquartile range. Molecular testing was not performed systematically throughout the study period; results are therefore presented descriptively. Mitotic count refers to the unit reported in the original pathology report (50 HPF or 5 mm^2^); no mathematical conversion was performed. AFIP risk classification was assigned according to the Miettinen and Lasota criteria based on tumor site, tumor size, and mitotic activity. Because the microscope field area represented by 50 HPF in the historical reports could not be verified, some risk-category misclassification cannot be excluded. Risk classification was considered not assessable in patients who had received neoadjuvant imatinib. Data are presented as median (IQR) or *n* (%). No formal hypothesis testing was performed.

**Table 2:** Surgical approach and operative characteristics of patients with primary localized gastric GIST.

Variable	Early Era 2015–2021 (*n* = 29)	Later Era 2022–2025 (*n* = 15)	Overall Cohort (*n* = 44)
**Final surgical approach**			
Completed laparoscopic, *n* (%)	18 (62.1)	4 (26.7)	22 (50.0)
Completed robotic, *n* (%)	0 (0)	6 (40.0)	6 (13.6)
Primary open, *n* (%)	8 (27.6)	5 (33.3)	13 (29.5)
Conversion to open, *n* (%)	3 (10.3)	0 (0)	3 (6.8)
**Extent of gastric resection**			
Local excision or wedge resection, *n* (%)	22 (75.9)	10 (66.7)	32 (72.7)
Segmental or partial gastric resection, *n* (%)	1 (3.4)	2 (13.3)	3 (6.8)
Distal or subtotal gastrectomy, *n* (%)	4 (13.8)	2 (13.3)	6 (13.6)
Total, proximal, or esophagogastric resection, *n* (%)	2 (6.9)	1 (6.7)	3 (6.8)
GIST-related multivisceral resection, *n* (%)	3 (10.3)	1 (6.7)	4 (9.1)
**Operative characteristics**			
Operative time, min, median (IQR)	69 (53–164)	75 (56–122.5)	73.5 (52.8–138.3)
R0 resection, *n* (%)	29 (100)	15 (100)	44 (100)
Tumor rupture, *n* (%)	0 (0)	0 (0)	0 (0)

Among 25 laparoscopically initiated procedures, 3 required conversion to open surgery, corresponding to an overall conversion rate of 12.0%. All conversions occurred in the early era. Abbreviations: GIST, gastrointestinal stromal tumor; IQR, interquartile range. Data are presented as median (IQR) or n (%). No formal hypothesis testing was performed.

**Table 3:** Perioperative outcomes after curative-intent resection of primary localized gastric GIST.

Variable	Early Era 2015–2021 (*n* = 29)	Later Era 2022–2025 (*n* = 15)	Overall Cohort (*n* = 44)
Length of hospital stay, days, median (IQR)	8 (6–10)	6 (4.5–8)	7.5 (5–9)
Any postoperative complication, *n* (%)	10 (34.5)	4 (26.7)	14 (31.8)
Clavien–Dindo grade I–II, *n* (%)	6 (20.7)	2 (13.3)	8 (18.2)
Clavien–Dindo grade ≥III, *n* (%)	4 (13.8)	2 (13.3)	6 (13.6)
30-day reoperation, *n* (%)	1 (3.4)	0 (0)	1 (2.3)
30-day mortality, *n* (%)	1 (3.4)	0 (0)	1 (2.3)

Abbreviations: GIST, gastrointestinal stromal tumor; IQR, interquartile range. Data are presented as median (IQR) or *n* (%). No formal hypothesis testing was performed.

**Table 4:** Case-level characteristics of the six robotic gastric GIST resections.

Case (Year)	Tumor Location	Pathological Size (cm)	Growth Pattern	Gastric Resection	Anatomical/Technical Context
1 (2022)	Anterior wall, lesser-curvature side	2.5	Predominantly endoluminal/intramural; no serosal extension on EUS	Local full-thickness excision with sutured closure	Localized lesion suitable for organ-preserving excision and intracorporeal closure
2 (2023)	Antrum, lesser-curvature side	4.4	Predominantly exophytic/extragastric; minor submucosal component	Segmental gastric resection	Organ-preserving resection of an exophytic antral tumor
3 (2023)	Greater curvature	3.8	Presumed predominantly exophytic/extragastric; no endoluminal lesion on gastroscopy	Wedge resection	Perigastric lesion adjacent to the greater curvature, suitable for wedge resection
4 (2024)	Antrum, posterior wall/greater-curvature side	3.1	Predominantly endoluminal/intramural submucosal growth	Wedge resection	Organ-preserving wedge resection of a posterior-wall antral lesion
5 (2024)	Cardia/fundus, greater-curvature side	6.1	Predominantly intramural/endophytic; expansile, non-infiltrative growth	Partial thoracoabdominal esophagectomy	Complex resection and reconstruction at the cardia/gastroesophageal junction
6 (2025)	Gastric body, posterior wall	2.2	Presumed predominantly exophytic/extragastric; no endoluminal lesion on gastroscopy	Wedge resection	CT-based localization between the stomach and pancreatic tail; wedge resection planned after multidisciplinary review

Abbreviations: EUS, endoscopic ultrasonography; GIST, gastrointestinal stromal tumor. Notes: Growth pattern was reconstructed retrospectively from imaging, endoscopic or endosonographic findings, operative documentation, and pathology reports. A separate case-specific rationale for choosing a robotic rather than laparoscopic approach was not documented prospectively. In institutional practice, robotic surgery was considered when the anticipated anatomy and required resection appeared suitable for a minimally invasive robotic approach and when the robotic platform was available. The anatomical and technical contexts shown in the table are therefore retrospective descriptions and should not be interpreted as predefined selection criteria.

## Data Availability

The data presented in this study are available from the corresponding author upon reasonable request and subject to the necessary institutional and data-protection approvals. The data are not publicly available due to patient privacy and data-protection restrictions.

## Supplementary Materials

The following supporting information can be downloaded at https://www.mdpi.com/article/10.3390/cancers18162562/s1. Table S1: Selection of the main study cohort from the institutional GIST database; Table S2: Postoperative complications within 30 days after surgery in the main cohort; Table S3: Institutional robot-assisted procedures involving GIST after introduction of the robotic program.

## Abbreviations

AFIP	Armed Forces Institute of Pathology
ASA	American Society of Anesthesiologists
BMI	body mass index
ECOG	Eastern Cooperative Oncology Group
GIST	gastrointestinal stromal tumor
HPF	high-power field
IQR	interquartile range
