# Supplementary material for: Surgical Approaches for Primary Gastric GIST After the Introduction of Robotic Surgery: A 10-Year Single-Center Experience
Source: Cancers (Basel). 2026 Aug 10;18(16):2562. doi: 10.3390/cancers18162562 (PMC13511205; doi:10.3390/cancers18162562)
Supplement: Supplementary file 1 [file cancers-18-02562-s001.zip › Table_S1_Cohort_Selection.pdf]

Surgical Approaches for Primary Gastric GIST After the Introduction of Robotic Surgery: A 10-Year Single-Center Experience

Julia Michel, Jens Hoeppner, Michael Leitz, Fabian Nimczewski, Zsolt Madarasz

Department of Surgery, Bielefeld University, Medical School and University Medical Center OWL, Campus Hospital Lippe, Detmold, Germany

Corresponding author: Julia Michel, Julia.Michel@klinikum-lippe.de

Table S1. Selection of the main study cohort from the institutional GIST database

Selection step	n	Reason for exclusion or handling
Unique patients recorded in the institutional GIST database during the study period	95	Institutional GIST population
**Non-gastric, multifocal, extra-gastrointestinal, or unknown primary GIST excluded**	**26**	The present study was restricted to gastric GIST; subgroup distribution is shown below
Jejunal or ileal GIST	*18*	*Non-gastric primary site*
Duodenal GIST	*1*	*Non-gastric primary site*
Colonic GIST	*2*	*Non-gastric primary site*
Rectal GIST	*1*	*Non-gastric primary site*
Extra-gastrointestinal GIST	*1*	*Extra-gastrointestinal origin*
Multifocal GIST involving more than one gastrointestinal site	*2*	*No single gastric primary site*
Unknown primary site	*1*	*Primary site could not be determined*
Patients with gastric GIST	69	Gastric primary site documented
Gastric GIST identified or resected during major surgery performed primarily for another disease	21	These cases were not considered GIST-directed surgical procedures because the primary indication for surgery was a non-GIST condition and perioperative outcomes could not be attributed specifically to the GIST procedure
Patients undergoing GIST-directed surgical treatment for gastric GIST	48	Gastric GIST constituted the primary indication for surgical treatment
Metastatic or palliative gastric GIST excluded from the main analysis	4	Excluded from the primary localized curative-intent cohort
**Final main cohort**	**44**	**Primary localized gastric GIST undergoing curative-intent resection**

Abbreviation: GIST, gastrointestinal stromal tumor.
