# Supplementary material for: Surgical Approaches for Primary Gastric GIST After the Introduction of Robotic Surgery: A 10-Year Single-Center Experience
Source: Cancers (Basel). 2026 Aug 10;18(16):2562. doi: 10.3390/cancers18162562 (PMC13511205; doi:10.3390/cancers18162562)
Supplement: Supplementary file 1 [file cancers-18-02562-s001.zip › Table_S2_Complications.pdf]

Surgical Approaches for Primary Gastric GIST After the Introduction of Robotic Surgery: A 10-Year Single-Center Experience

Julia Michel, Jens Hoeppner, Michael Leitz, Fabian Nimczewski, Zsolt Madarasz

Department of Surgery, Bielefeld University, Medical School and University Medical Center OWL, Campus Hospital Lippe, Detmold, Germany

Corresponding author: Julia Michel, Julia.Michel@klinikum-lippe.de

Table S2. Postoperative complications within 30 days after surgery in the main cohort

Case no.	Era	Surgical procedure	Postoperative complication(s)	Management	Clavien–Dindo grade	30-day reoperation	30-day mortality
1	Early	Open wedge resection	Transient ileus symptoms and urinary retention	Non-operative management; details not further specified	II*	No	No
2	Early	Open wedge resection	Postoperative bleeding from the laparotomy wound	Bedside suture ligation without general anesthesia	IIIa	No	No
3	Early	Laparoscopic atypical partial gastric resection	Infected postoperative fluid collection/hematoma in the left upper abdomen	Antibiotic therapy and ultrasound-guided percutaneous drainage with subsequent daily irrigation	IIIa	No	No
4	Early	Open subtotal gastric resection with Roux-en-Y reconstruction	Allergic exanthem	Antihistamine treatment	II	No	No
5	Early	Laparoscopic wedge resection	Transient increase in inflammatory markers	Clinical observation and ultrasonographic assessment	I	No	No
6	Early	Laparoscopic subtotal gastric resection with Roux-en-Y reconstruction	Cardiopulmonary arrest with hemodynamic instability and seizure	Resuscitation, vasopressor therapy, and intensive care treatment	IVb	No	No
7	Early	Open wedge resection with splenectomy	Postoperative anemia requiring transfusion	Blood transfusion	II	No	No
8	Early	Open local gastric excision	Gastric suture-line leak with sepsis	Surgical revision and intensive care treatment	V	Yes	Yes

Case no.	Era	Surgical procedure	Postoperative complication(s)	Management	Clavien–Dindo grade	30-day reoperation	30-day mortality
9	Early	Laparoscopic wedge resection	Allergic reaction to piperacillin	Antihistamine treatment	II	No	No
10	Early	Open total gastrectomy	Postoperative anemia and mild pancreatitis	Transfusion of 1 unit of packed red blood cells and conservative treatment	II	No	No
11	Later	Robotic local excision with primary closure	Postoperative intraluminal bleeding	Endoscopic hemostasis without general anesthesia	IIIa	No	No
12	Later	Open wedge resection	Wound-healing disorder with wound dehiscence	Wound opening and irrigation without general anesthesia	IIIa	No	No
13	Later	Open subtotal gastrectomy	Postoperative anemia requiring transfusion	Blood transfusion	II	No	No
14	Later	Robotic wedge resection	Postoperative fever	Antibiotic therapy with piperacillin/tazobactam	II	No	No

Data are presented at the patient level. When more than one postoperative complication occurred, the highest Clavien–Dindo grade is shown. *Case 1 had both grade I and grade II events; grade II is reported as the highest grade.
