# Supplementary material for: Surgical Approaches for Primary Gastric GIST After the Introduction of Robotic Surgery: A 10-Year Single-Center Experience
Source: Cancers (Basel). 2026 Aug 10;18(16):2562. doi: 10.3390/cancers18162562 (PMC13511205; doi:10.3390/cancers18162562)
Supplement: Supplementary file 1 [file cancers-18-02562-s001.zip › Table_S3_Robotic_Activity.pdf]

Surgical Approaches for Primary Gastric GIST After the Introduction of Robotic Surgery: A 10-Year Single-Center Experience

Julia Michel, Jens Hoeppner, Michael Leitz, Fabian Nimczewski, Zsolt Madarasz

Department of Surgery, Bielefeld University, Medical School and University Medical Center OWL, Campus Hospital Lippe, Detmold, Germany

Corresponding author: Julia Michel, Julia.Michel@klinikum-lippe.de

Table S3. Institutional robot-assisted procedures involving GIST after introduction of the robotic program

Clinical context	2022	2023	2024	2025*	Total
GIST-directed robot-assisted resection of gastric GIST	1	2	2	1	6
Robot-assisted major surgery for another disease with synchronous gastric GIST resection	0	3	0	0	3
Robot-assisted procedures involving non-gastric GIST	0	2	1	0	3
**All robot-assisted procedures involving GIST**	**1**	**7**	**3**	**1**	**12**

*January to June 2025. GIST, gastrointestinal stromal tumor. Synchronous gastric GIST resections performed during major surgery for another disease and procedures involving non-gastric GIST were not included in the perioperative outcome analysis of the main cohort. The three robot-assisted procedures involving non-gastric GIST comprised two colorectal GIST resections and one small-bowel GIST resection.
